# Comparison of the Photosynthetic Capacity of *Phragmites australis* in Five Habitats in Saline‒Alkaline Wetlands

**DOI:** 10.3390/plants9101317

**Published:** 2020-10-06

**Authors:** Subang An, Xingtu Liu, Bolong Wen, Xiaoyu Li, Peng Qi, Kun Zhang

**Affiliations:** 1 Key Laboratory of Wetland Ecology and Environment, Northeast Institute of Geography and Agroecology, Chinese Academy of Sciences, Changchun 130102, China; ansubang@iga.ac.cn (S.A.); wenbolong@iga.ac.cn (B.W.); lixiaoyu@iga.ac.cn (X.L.); qipeng@iga.ac.cn (P.Q.); 2 University of Chinese Academy of Sciences, Beijing 100049, China; 3 College of Wetland Science, Southwest Forestry University, Kunming 650224, China; zhangkun@swfu.edu.cn

**Keywords:** Amur River Basin, Heilongjiang Province, Jilin Province, *Phragmites australis*, wetlands, farmland, paddy, photosynthesis, biomass

## Abstract

Water shortages have an important impact on the photosynthetic capacity of *Phragmites australis*. However, this impact has not been adequately studied from the perspective of photosynthesis. An in-depth study of the photosynthetic process can help in better understanding the impact of water shortages on the photosynthetic capacity of *P. australis*, especially on the microscale. The aim of this study is to explore the photosynthetic adaptation strategies to environmental changes in saline‒alkaline wetlands. The light response curves and CO_2_ response curves of *P. australis* in five habitats (hygrophilous, xerophytic, psammophytic, abandoned farmland, paddy field drainage) in saline‒alkaline wetlands were measured at different stages of their life history, and we used a nonrectangular hyperbolic model to fit the data. It was concluded that *P. australis* utilized coping strategies that differed between the growing and breeding seasons. *P. australis* in abandoned farmland during the growing season had the highest apparent quantum efficiency (AQE) and photosynthetic utilization efficiency for weak light because of the dark environment. The dark respiration rate of *P. australis* in the drainage area of paddy fields was the lowest, and it had the highest values for photorespiration rate, maximum photosynthetic rate (Pmax), photosynthetic capacity (Pa), biomass, maximum carboxylation rate (Vcmax), and maximum electron transfer rate (Jmax). The light insensitivity of *P. australis* increased with the transition from growing to breeding season, and the dark respiration rate also showed a downward trend. Moreover, Vcmax and Jmax would decline when Pmax and Pa showed a declining trend, and vice versa. In other words, Vcmax and Jmax could explain changes in the photosynthetic capacity to some extent. These findings contribute to providing insights that Vcmax and Jmax can directly reflect the variation in photosynthetic capacity of *P. australis* under water shortages in saline‒alkaline wetlands and in other parts of world where there are problems with similarly harmful environmental conditions.

## 1. Introduction

Vegetation is a fundamental part of wetlands, so it is important to study the photosynthetic response mechanisms of vegetation for wetland protection [1,2]. *Phragmites australis* is one of the typical wetland plants and, therefore, studying the photosynthetic process of *P. australis* in differing environments is helpful in further understanding the response strategies of plants to different environmental conditions [3,4].

The physiological characteristics of photosynthetic carbon fixation in plants are mainly studied in terms of the effects of water (water level), salt [5], and heavy metal stress [6] on photosynthesis and fluorescence in plants. Combined with the plant community characteristics and growth characteristics, models are used to fit the photosynthetic carbon fixation process [7,8]. For example, the tolerance to flooding of four species, including *P. australis*, has been compared using isotopic techniques. The results showed that *P. australis* was the most tolerant plant because flooding resulted in an increase in the stomatal conductance of *P. australis*, and anaerobic enzymes in the rhizosphere improved its tolerance; in addition, the high photosynthetic rate (Pa) contributed to biomass accumulation and CO_2_ fixation during this period [9]. *P. australis* is more tolerant to short-term flooding than to high salinity [10] because these variables affect chemical oxygen demand (COD) and other indicators in water. The photosynthetic rate decreases with increases in chemical oxygen demand, and higher COD can interfere with plant metabolism [11]. Some plants can improve their tolerance to a high-salinity and heavy metal environment by secreting protective enzymes such as superoxide dismutase (SOD), but this only applies under low-salinity conditions; under high salinity, the ability of plants to produce SOD will decrease or even disappear, thus inhibiting plant growth due to reduced protection against heavy metal toxicity [12]. Moreover, high salinity stress also has a great influence on the chlorophyll content of plant leaves, leading to a decrease in Pa. At the same time, the decrease in Pa under low-salinity stress is related to stomatal closure, but after exceeding the concentration threshold, stomatal closure is no longer the main reason for the decrease in Pa [13].

The effect of the water level on plant photosynthesis is mainly reflected in the distribution of biomass in roots, stems, and leaves as well as the photosynthetic process. Generally, in the early growing season, the biomass is mainly distributed to the leaves and stems to facilitate photosynthetic carbon sequestration. In the middle of the growing season, it is mainly distributed to the stems, while in the late growing season, it is distributed to the roots. Under adequate water conditions, plants will further reduce the allocation of biomass to the roots and increase the allocation of biomass to the stems and leaves. However, in the case of water shortage, the biomass allocation in leaves will be reduced while being increased in roots [14]. As for the photosynthetic process, at different growth or breeding stages, the response of plant photosynthetic carbon sequestration characteristics to hydrological conditions also differs; by adjusting the stomatal conductance of leaves and its photosynthetic rate [15], plants can adapt to changes in water depth and evolve into different ecological types. Therefore, it is necessary to study the effects of hydrological conditions on the photosynthesis of plants in combination with biomass.

The carbon sources and sink functions of saline‒alkaline wetlands, as a special type of inland wetland, differ from those of freshwater wetlands. In China, the Western Songnen Plain is one of the main distribution areas for saline‒alkaline wetlands. According to the results of China’s second national survey of wetland resources, there are 430 km^2^ of wetlands in Western Jilin Province, with reed marshes being dominant. Niuxintaobao Wetland is one of the typical distribution areas for reed marshes in the Western Songnen Plain. It is also a typical saline‒alkaline reed marsh, with abundant reed resources, with a vegetation coverage rate of around 85% [16]. There is a distinct water gradient from the center to the shore in the Niuxintaobao Wetland, and the change in water gradient results in different reed habitats. Therefore, it is important to understand the photosynthetic adaptation strategies of *P. australis* to environmental changes in saline‒alkaline wetlands. As such, based on model fitting, the two main objectives of this study were to (1) characterize the photosynthetic characteristics of *P. australis* in different environments and (2) discuss how *P. australis* responds to environmental changes on the microscale (for both light-dependent and -independent reactions). We anticipate that the findings from this study will help in further understanding the relationship between plants and the environment.

## 2. Results

### 2.1. Characteristics of Light Response Curve

#### 2.1.1. Characteristics of Light Response Curve in the Growing Season

**Figure 1:**
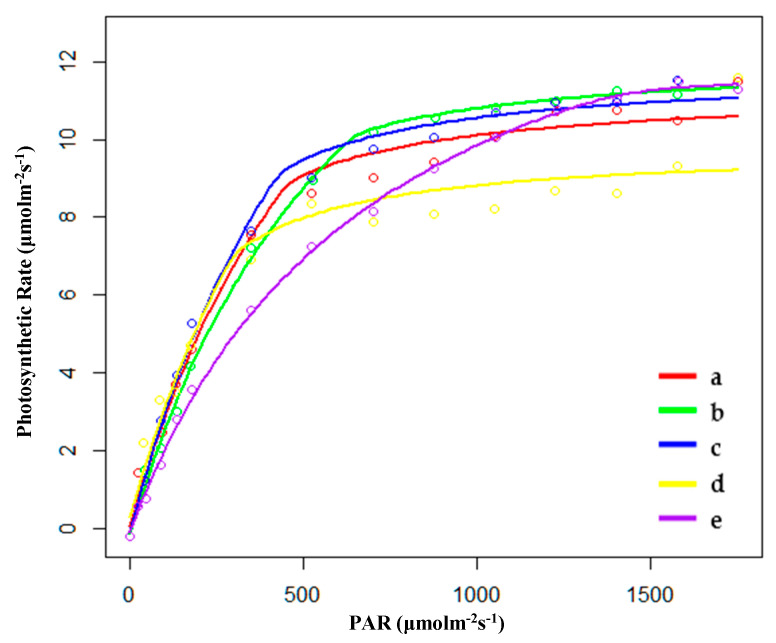
Fitting results of light response curves of (**a**) hygrophilous type of *P. australis*, (**b**) xerophytic type of *P. australis*, (**c**) psammophytic type of *P. australis*, (**d**) *P. australis* in abandoned farmland, and (**e**) *P. australis* in paddy field drainage during the growing season. PAR: photosynthetically active radiation.

During the growing season, the saturated light intensity (Im) of HP (hygrophilous type of *P. australis*) was the lowest, and the Im of PP (*P. australis* in drainage area of paddy field) was the highest. The apparent quantum efficiency (AQE) of FP (*P. australis* in abandoned farmland) had the highest value, indicating that *P. australis* in abandoned farmland had the highest photosynthetic efficiency under low light conditions. The value for maximum net photosynthetic rate (Pmax) of PP was the highest, and its biomass (Bm) per unit area was also higher than that of other habitats (Table 1 and Figure 1). The rate of dark respiration (Rd) of PP was also the lowest among the five habitats. Thus, higher Pmax and lower Rd appear to be beneficial for the accumulation of biomass in the growth stage.

#### 2.1.2. Characteristics of Light Response Curve in the Breeding Season

**Figure 2:**
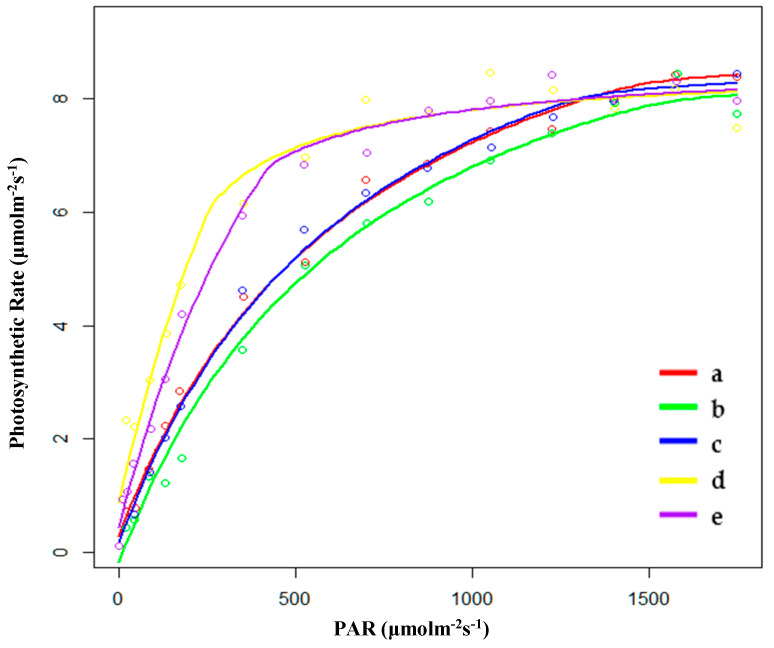
Fitting results of light response curves of (**a**) hygrophilous type of *P. australis*, (**b**) xerophytic type of *P. australis*, (**c**) psammophytic type of *P. australis*, (**d**) *P. australis* in abandoned farmland, and (**e**) *P. australis* in paddy field drainage during the breeding season. PAR: photosynthetically active radiation.

During the breeding season, the saturated light intensity (Im) of PP was the lowest, and the Im of FP was the highest. There were no significant differences in the rate of dark respiration (Rd) between HP (hygrophilous type of *P. australis*) and XP (xerophytic type of *P. australis*). SP (psammophytic type of *P. australis*) had the lowest biomass. The order of AQE was FP < HP < XP < PP < SP, and the order of Pmax was FP < PP < HP < XP < SP. SP had the highest values for AQE and Pmax. Furthermore, SP also had the lowest Rd. However, PP still had the largest biomass (Table 2 and Figure 2). Therefore, there may be other factors that determined the accumulation of biomass in the stage of breeding. At the same time, after entering the breeding season, the change in Pmax varied depending on the habitat. The Pmax of SP and PP changed greatly. The Pmax of SP increased by around 50% while the Pmax of PP decreased by 50.5%. However, the Pmax of the others did not change significantly; the Pmax of HP and FP decreased while that of XP increased.

### 2.2. Characteristics of CO_2_ Response Curve

#### 2.2.1. Characteristics of CO_2_ Response Curve in the Growing Season

**Figure 3:**
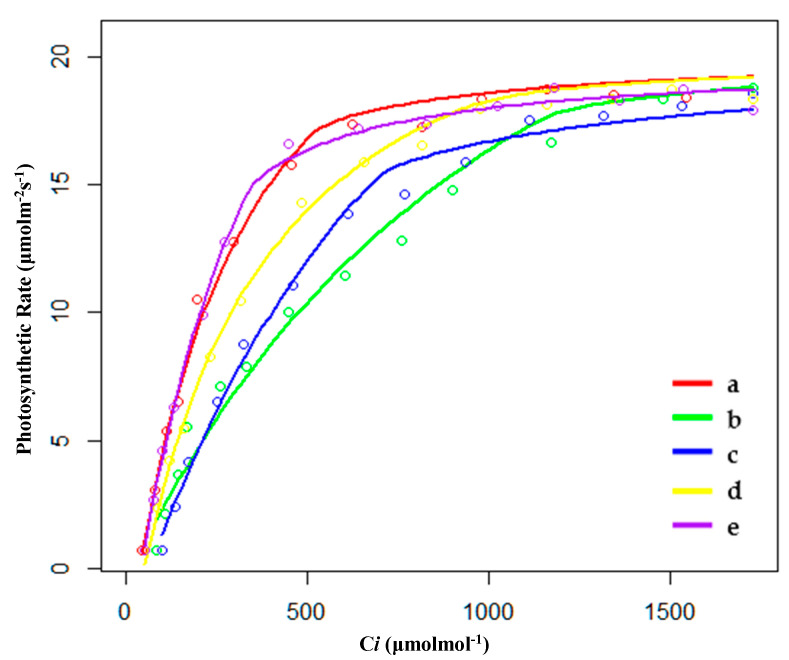
Fitting results of CO_2_ response curves of (**a**) hygrophilous type of *P. australis*, (**b**) xerophytic type of *P. australis*, (**c**) psammophytic type of *P. australis*, (**d**) *P. australis* in abandoned farmland, and (**e**) *P. australis* in paddy field drainage during the growing season. C*i*: intercellular CO_2_ concentration.

During the growing season, the order of CO_2_ saturation point (Cm) was PP < SP < XP < HP < FP; the order of CO_2_ compensation point (Cc) was SP < PP < XP < HP < FP (Table 3 and Figure 3). FP had the highest Cm and Cc, indicating that FP could make use of a wide range of concentrations of CO_2_. However, the quantum efficiency of CO_2_ (φCO_2_) of FP was the lowest, indicating that the utilization efficiency of low-concentration CO_2_ for FP was lower than compared to other conditions; this may be why FP needed to make use of a wide range of concentrations of CO_2_. PP had the highest value of φCO_2_, and its rate of respiration (Rl) and photosynthetic capacity (Pa) were also the largest among the five habitats. However, as mentioned before, PP still had the largest biomass; a higher respiratory rate would not be enough to affect the biomass accumulation.

#### 2.2.2. Characteristics of CO_2_ Response Curve in the Breeding Season

**Figure 4:**
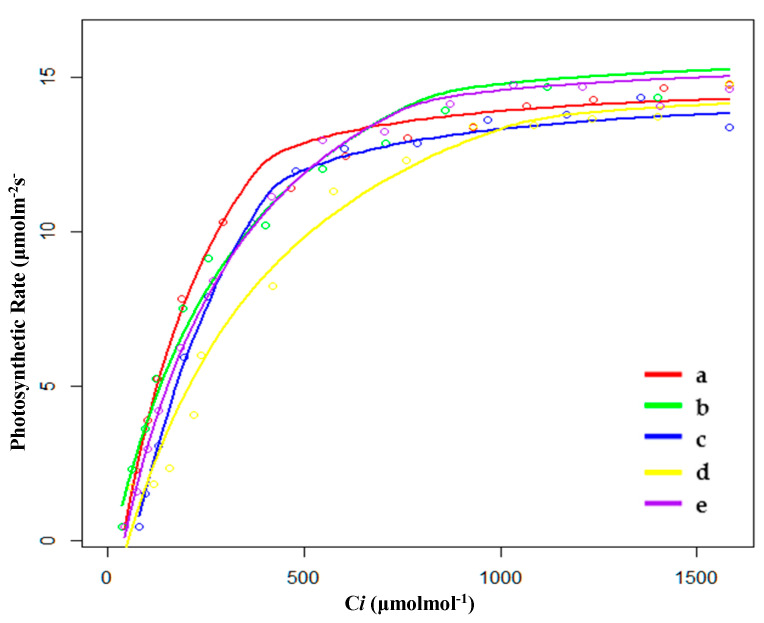
Fitting results of CO_2_ response curves of (**a**) hygrophilous type of *P. australis*, (**b**) xerophytic type of *P. australis*, (**c**) psammophytic type of *P. australis*, (**d**) *P. australis* in abandoned farmland, and (**e**) *P. australis* in paddy field drainage during the breeding season. C*i*: intercellular CO_2_ concentration.

During the breeding season, the order of CO_2_ saturation point (Cm) was SP < PP < XP < HP < FP, and the order of CO_2_ compensation point (Cc) was SP < HP < XP < PP < FP (Table 4 and Figure 4); hence, the values of Cm and Cc were still the highest for FP. Moreover, the quantum efficiency of CO_2_ (φCO_2_) of FP was also the lowest, indicating that FP still needed to make use of a wide range of CO_2_ concentrations. The photosynthetic capacity (Pa) was similar to that of the growing season, i.e., under conditions of sufficient light and CO_2_, *P. australis* showed strong photosynthetic capacity in five habitats, especially in the drainage area of paddy field (PP). However, compared with the growth period, the photosynthetic capacity (Pa) of PP, HP, and FP decreased, while that of XP and SP increased.

## 3. Discussion

### 3.1. Photosynthetic Characteristics of P. australis in the Growing Season

The fitting results for the light response curve demonstrated that apparent quantum efficiency (AQE) was one of the most important indicators for characterizing the ability of plants to assimilate CO_2_ under low light conditions. The slope of the light response curve at the weak light stage (i.e., the smaller PAR value interval) was calculated as the apparent quantum efficiency, which means the average amount of CO_2_ assimilated by one photon [17,18]. During the growing season, the AQE of FP was the highest (0.047 ± 0.004) in five habitats, indicating that FP had a strong ability to utilize weak light. Field investigations also revealed that in abandoned farmland, *P. australis* mainly grew in a shady environment (shading by trees is one of the reasons leading to the abandonment of farmland). Furthermore, the high AQE also indicated that *P. australis* adapted to long-term shading, indicating that it had higher photosynthetic efficiency in weak light. The rate of respiration represents the rate at which plants consume organic matter. It is generally believed that a higher respiration rate is not conducive to the accumulation of organic matter. Maximum photosynthetic rate (Pmax) represents the ability to assimilate CO_2_ under sufficient light; the higher the value of Pmax, the higher the rate of carbon sequestration, and the more favorable it is for the accumulation of organic matter [19,20]. PP had the highest Pmax and the lowest respiration rate. Therefore, the general rule of biomass accumulation of *P. australis* in five habitats was as follows: the higher the value of Pmax, the larger the biomass. Studies have found that rich soil nutrient content is conducive to the accumulation of photosynthetic carbon sequestration of plants [21,22]. The reason for PP’s greater Pmax is presumed to be related to the use of fertilizers in paddy fields, which indirectly results in higher N, P, and other nutrient elements in soil than in other habitats, which is more conducive to the fixation of CO_2_ and accumulation of organic matter [23]. However, PP had the lowest Pmax. Some studies have shown that *P. australis* can regulate its genes, to some extent, to adapt to high-salinity environments. These genetic regulations include higher relative expression levels of genes associated with photosynthesis and lignan biosynthesis, indicative of a greater ability to maintain growth under saline conditions [24]. At the same time, the distribution of photosynthetically fixed C in roots and soils also changes, for example, with lower contents of photosynthetically fixed C in roots and higher contents in soil [25].

Photosynthetic capacity (Pa) is one of the most important indicators for analyzing the characteristics of the CO_2_ response curve. It is used to characterize the maximum potential of fixing CO_2_ under conditions of sufficient light and CO_2_. Photorespiration refers to the consumption of superfluous substances by respiration when high amounts of [H] and ATP accumulate in the photoreaction but the photosynthetic dark reaction is inhibited so as to prevent their accumulation, affecting plant metabolism [26,27,28,29]. Therefore, the rate of photorespiration (Rl) in plants can reflect their photoreaction rate to a certain extent, and this then affects the final net photosynthetic rate. During the growing season, the general rule for the photosynthetic capacity of *P. australis* in the five studied habitats was that the higher the Rl value, the higher the Pa value. The Rl of PP was the highest, and the Pa of PP was also the highest among the five habitats. Moreover, the CO_2_ quantum efficiency (φCO_2_) of PP was also the highest, indicating that it had the highest photosynthetic efficiency for low concentrations of CO_2_.

**Figure 5:**
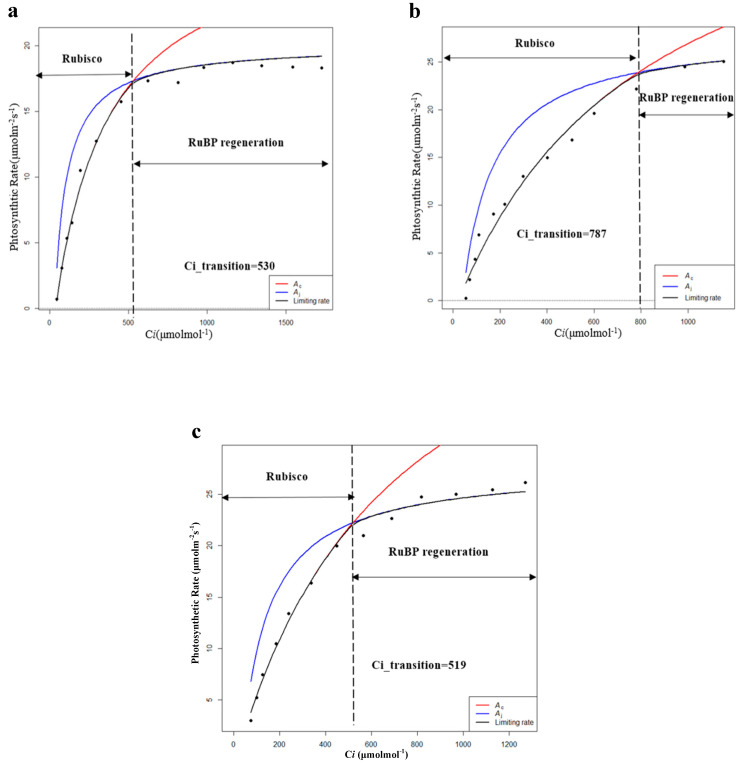

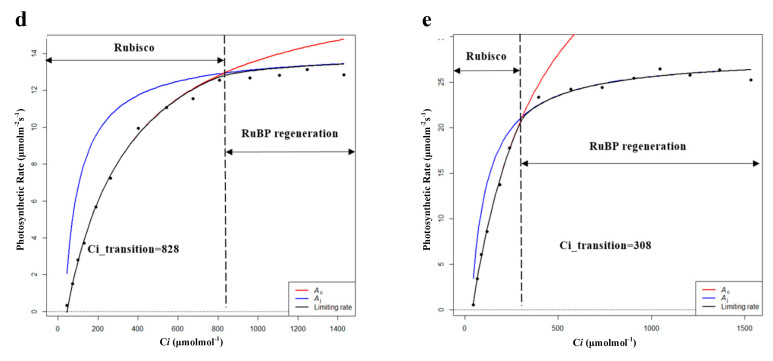
Modeling results of photosynthetic rate limitations of (**a**) hygrophilous type of *P. australis*, (**b**) xerophytic type of *P. australis*, (**c**) psammophytic type of *P. australis*, (**d**) *P. australis* in abandoned farmland, and (**e**) *P. australis* in paddy field drainage during the growing season. *A_c_* is the gross photosynthetic rate when Rubisco activity is limiting; *A_j_* is the gross photosynthetic rate when RuBP regeneration is limiting (RuBP: ribulose bisphosphate; C*i*: intercellular CO_2_ concentration).

By further fitting the CO_2_ response curve, the limits for the photosynthetic rate in the dark reaction process were obtained for different intercellular CO_2_ concentrations (Ci). Vc represents the limitation of Rubisco carboxylase and J represents the limitation of RuBP (ribulose bisphosphate) regeneration. Therefore, the intersection point (Ci_transition) of the Vc-limit curve (blue) and J-limit curve (red) was the demarcation between the limitation of Rubisco carboxylase and the limitation of RuBP regeneration. When C < Ci_transition, the photosynthetic rate is mainly limited by Vc, and when C > Ci_transition, the photosynthetic rate is mainly limited by J [30,31]. According to the fitting results, during the growing season, the Vcmax and Jmax of PP were the highest among the five habitats, and the photosynthetic capacity (Pa) of PP was also the highest (Table 5 and Figure 5). Moreover, the value of Ci_transition of PP was 308 ppm, which was lower than the general environmental CO_2_ concentration (around 400 ppm). Therefore, the photosynthetic rate of PP was mainly determined by Vc and J, while the photosynthetic rates of others were mainly determined by Vc.

### 3.2. Photosynthetic Characteristics of P. australis in the Breeding Season

By comparing the characteristics of light response curves, reeds in all habitats showed an increase in the value of the light saturation point (Im) and a decrease in the value of AQE after entering the breeding season, which meant a gradual adaptation to and utilization of the high-light environment. It was believed that the general downward trend observed for the value of AQE during the process of plant growth may be related to the increase in average solar radiation intensity [32]. Meanwhile, the rates of dark respiration of *P. australis* in all habitats were lower than those of the growing season, and the biomass showed accumulation with a decrease in the rates of dark respiration. Moreover, the rate of biomass accumulation of FP was the highest among the five habitats (386.7%). However, the Pmax of HP, FP, and PP showed a downward trend. The decrease in photosynthetic rate was related to the decrease in stomatal conductance, and the decrease in stomatal conductance was related to the increase in salinity [33]. Some studies have shown that reeds could adapt to a saline and alkaline environment by rapid ecological evolution and phenotypic differentiation. At the same time, reeds could also adapt to a harsh environment by reducing the photosynthetic rate or chlorophyll concentration and increasing the K^+^ concentration in leaves [34,35,36].

**Figure 6:**
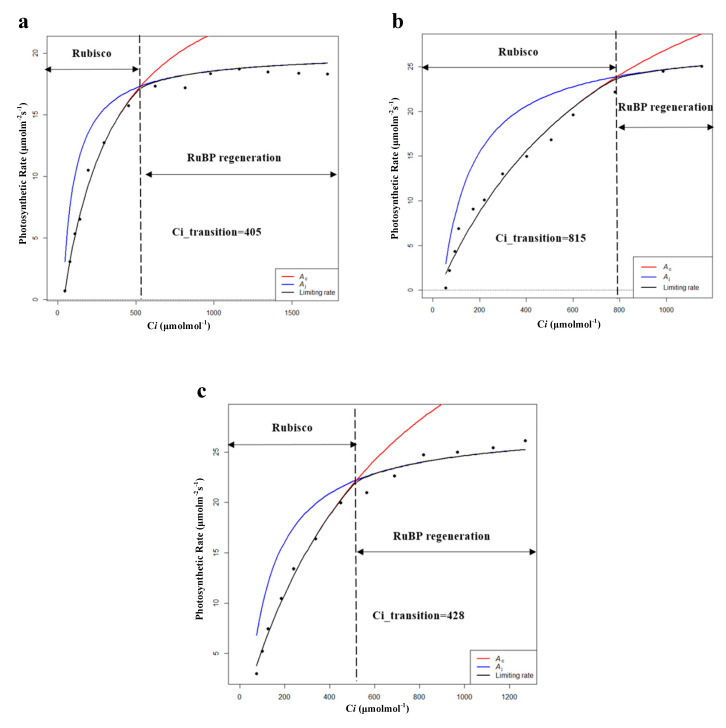

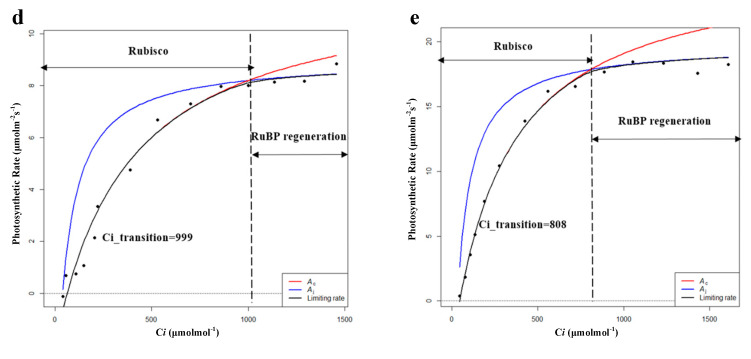
Modeling results of photosynthetic rate limitations of (**a**) hygrophilous type of *P. australis*, (**b**) xerophytic type of *P. australis*, (**c**) psammophytic type of *P. australis*, (**d**) *P. australis* in abandoned farmland, and (**e**) *P. australis* in paddy field drainage during the breeding season. *A_c_* is the gross photosynthetic rate when Rubisco activity is limiting; *A_j_* is the gross photosynthetic rate when RuBP regeneration is limiting (RuBP: ribulose bisphosphate; C*i*: intercellular CO_2_ concentration).

By comparing the characteristics of CO_2_ response curves, reeds in all habitats showed a decrease in the value of CO_2_ quantum efficiency (φCO_2_) after entering the breeding season, which represents an adaptation to high concentrations of CO_2_. Except for HP, reeds in all habitats showed an increase in the value of CO_2_ compensation points (Cc), which meant decreased photosynthetic sensitivity to low concentrations of CO_2_. In addition, PP had the highest φCO_2_, Rl, and Pa in both the growing and breeding seasons. Moreover, Vcmax and Jmax as well as Pmax and Pn of XP and SP showed an upward trend while showing a downward trend for HP, FP, and PP (Table 6 and Figure 6). The results showed that the fitting results of the light response curves and the CO_2_ response curves were consistent. It was also found that XP and SP entered the withering season later than HP, FP, and PP during the field investigation, which may be related to the later decline in ability to undergo dark reaction (Vcmax, Jmax) of XP and SP. Therefore, Vcmax and Jmax, as important indicators reflecting the characteristics of photosynthetic dark reaction, could explain the changes in photosynthetic rate to some extent [37]. However, Vcmax and Jmax represent only the dark reaction part of photosynthesis, and if combined with the chlorophyll fluorescence parameters, i.e., the characteristics of the light reaction part of photosynthesis, the variations in the photosynthetic rate will be explained more comprehensively. Hence, it is necessary to conduct further studies on the specific photosynthetic process of *P. australis*.

## 4. Materials and Methods

### 4.1. Study Area

**Figure 7:**
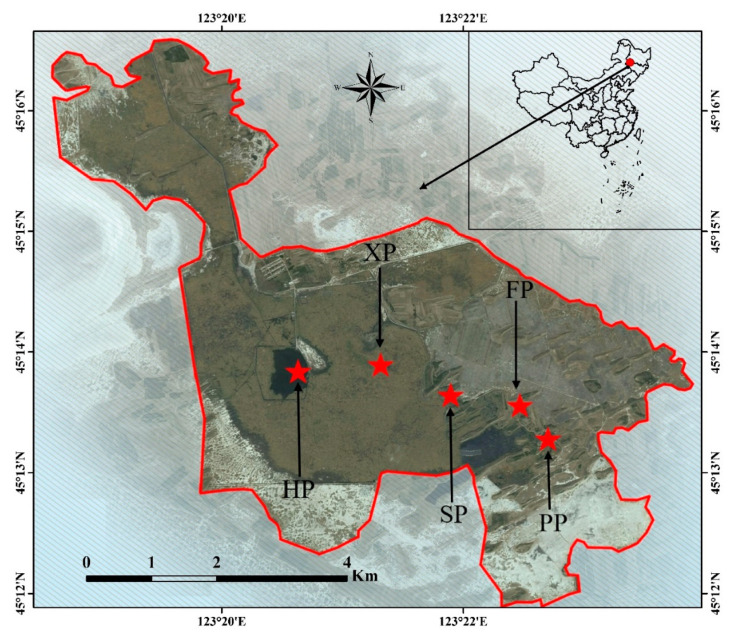
Location of the study area.

Niuxintaobao Wetland (45°13′–45°16′ N, 123°13′–123°21′ E) is located in the west of Songnen Plain in Northeastern China (Figure 7). Administratively, it is within the provinces of Jilin and Heilongjiang of China. It is formed by water accumulation in the interfluvial lowlands caused by the hydraulic movement of Huolin and Taoer Rivers. It is moderately saline‒alkaline, with an area of around 33 km^2^. The main source of water supply is Taoer River [38]. *P. australis* saline‒alkaline marshes are distributed in the study region, and it is characterized by a typical semiarid and moderate monsoon climate with distinctive seasons; the total annual sunlight is 5259 MJ/m^2^, the frost-free period is 137 d of the year [39], and it is one of the typical distribution areas of reeds in inland China. 

A field survey was carried out during May (growing season) and August (breeding season). Reed habitats were classified according to the measured soil moisture as follows: hygrophilous (HP), xerophytic (XP), psammophytic (SP), abandoned farmland (FP), or paddy field drainage (PP) [40]. Ten stands (5 m × 5 m) in each habitat were selected and used as replicates for all habitats (Table 7 and Figure 7).

### 4.2. Experimental Design

#### 4.2.1. Biomass Collection

There are non-destructive sampling methods using remote sensing spectroscopy for measuring plant biomass, and these methods are mainly used in the macro or large-scale research [41,42,43,44,45,46]. In order to directly reflect the characteristics of biomass, combined with the sampling methods commonly used by previous researchers [47,48,49,50,51,52], we chose the harvesting method to measure the biomass. That is, the aboveground parts of *P. australis* in five habitats were mowed in a 0.5 m × 0.5 m square and then dried in an oven at 75 °C for 48 h. The final weight was recorded when the weight showed no further reductions.

#### 4.2.2. Measurement of Light Response Curve

The third top leaves of 10 shoots from each stand were used as replicates. The relative humidity was 45–50% and the temperature was around 25 °C. The light response curve was measured by LI-6400XT (LICOR, Lincoln, NE, USA) at 9:00–11:00 on a bright, clear day in May and August. Full light induction was carried out after installing the red and blue light source leaf chamber (6400-02B). After successful induction, the stable photo values under 15 light intensity (PAR, μmol/m^2^/s) gradients (2000, 1800, 1600, 1400, 1200, 1000, 800, 600, 400, 200, 150, 100, 50, 25, and 0) were selected and recorded in the file. Photo values were recorded in order of light intensity, from high to low. The standard of photo value recording is that the intake concentration of the instrument is stable without leakage; the stomatal conductance (Cond), intercellular CO_2_ concentration (Ci), and transpiration rate (Tr) of line C are all positive, the value of Cond is between 0 and 1; and the change rate of photo value (△P) is less than 2%.

#### 4.2.3. Measurement of CO_2_ Response Curve

The leaves were the same as those used in measuring the light response curve. After full light induction, the CO_2_ mixer was used to control the CO_2_ concentration gradient of 2000, 1800, 1600, 1400, 1200, 1000, 800, 600, 400, 200, 150, 100, 50, 25, and 0. The saturated light intensity (2000 μmol/m^2^/s) was chosen as the light intensity. However, the photo values were recorded in the following order: 400, 200, 150, 100, 50, 25, 0, 400, 600, 800, 100, 1200, 1400, 1600, 1800, and 2000. The standard of recording the photo value is the same as that of the measurement of the light response curve.

### 4.3. Data Calculation and Analysis

#### 4.3.1. Fitting Light Response Curve

The fitting of light response curve is based on the nonrectangular hyperbolic model (Equation (1)) [53,54]:

(1) $$Pn\left(I\right)=\frac{aI+Pmax-\sqrt{{\left(aI+Pmax\right)}^{2}-4\theta aIPmax}}{2\theta }-Rd$$

where *Pn* is the photosynthetic rate, *I* is the light intensity, *a* is the apparent quantum efficiency (AQE), *Pmax* is the maximum photosynthetic rate, *Rd* is the respiratory rate, and *θ* is the correction coefficient. According to the formula, *Ic* is set as the light compensation point, i.e., the value of *I* when *Pn*(*I*) = 0, *Im* is the light saturation point, i.e., the value of *I* when *Pn*’(*I*) = 0, and *Pn’*(*I*) is the first derivative of the function *Pn*(*I*).

#### 4.3.2. Fitting the CO_2_ Response Curve

A nonrectangular hyperbolic model was also used to fit the CO_2_ response curve (Equation (2)) [55], but there are corresponding deformations when calculating *Vcmax* (represented by *Ac* in Equation (3)) and *Jmax* (represented by *Aj* in Equation (3)) [56]:

(2) $$Pn\left(C\right)=\frac{aC+Pa-\sqrt{{\left(aC+Pa\right)}^{2}-4\theta aCPa}}{2\theta }-Rl$$

where *Pn* is the photosynthetic rate, *C* is the CO_2_ concentration, *a* is the CO_2_ quantum efficiency (φCO_2_), *Pa* is the photosynthetic capacity, *Rl* is the respiratory rate, and *θ* is the correction coefficient. According to the formula, *Cc* is set as the CO_2_ compensation point, i.e., the value of *C* when *Pn*(*C*) = 0, *Cm* is the CO_2_ saturation point, i.e., the value of *C* when *Pn*’(*C*) = 0, and *Pn’*(*C*) is the first derivative of the function *Pn*(*C*).

(3) $$Am=\frac{Ac+Aj-\sqrt{{\left(Ac+Aj\right)}^{2}-4\theta AcAj}}{2\theta }-Rl$$

where *Am* is the hyperbolic minimum of *Ac* and *Aj*, *Ac* is the gross photosynthetic rate when Rubisco activity is limiting, *Aj* is the gross photosynthetic rate when RuBP regeneration is limiting, *Rl* is the respiratory rate, and *θ* is the correction coefficient.

#### 4.3.3. Statistical Analysis

The least squares method was used to estimate the fit of the experimental data. The test of fitting results could be divided into a goodness of fit test and a significance test for the regression equation. The decision coefficient *R^2^* was used to verify the goodness of fit, and the F test was used to verify the significance of the regression equation. One-way ANOVA was used to test the differences in photosynthetic characteristics of *P. australis* in different habitats. The confidence intervals of all the analyses were 95%. Statistical software SPSS22.0 for Windows (IBM Corp., Armonk, NY, USA) was used for the above statistical analyses, and the experimental data and regression model were also plotted and analyzed by R language software package “plantecophys”, written by Remko Duursma [57] (v.3.4.2; R Foundation for Statistical Computing, Vienna, Australia).

## 5. Conclusions

This study was the first attempt to compare the response of *P. australis* to environmental changes from the perspective of the photosynthetic process. The findings indicate that with the transition from the growing season to the breeding season, *P. australis* showed decreased photosynthetic sensitivity, the rate of dark respiration also showed a downward trend, and plants were more conducive to the accumulation of biomass. *P. australis* in the drainage area of a paddy field benefited from abundant nutrition; its biomass and photosynthetic capacity were the highest. Moreover, the maximum photosynthetic rate and photosynthetic capacity of *P. australis* in all five habitats had the same trend of variation, and the trend was consistent with that of Vcmax and Jmax. Overall, our results suggest that study of Vcmax and Jmax is beneficial for exploring the photosynthetic adaptation strategies to harsh environmental changes, such as water shortages in saline‒alkaline wetlands, and in other areas facing the same problems in the world. However, if combined with the chlorophyll fluorescence parameters, i.e., the characteristics of the light reaction part of photosynthesis, the variation in photosynthetic capacity can be explained more comprehensively. Hence, the specific photosynthetic process of *P. australis* deserves further research.

## Figures and Tables

**Table 1:** Photosynthetic physiological characteristics of *P. australis* in the growing season (Im: saturated light intensity; AQE: apparent quantum efficiency; Rd: rate of dark respiration; Pmax: maximum net photosynthetic rate; Bm: biomass; HP: hygrophilous type of *P. australis*; XP: xerophytic type of *P. australis*; SP: psammophytic type of *P. australis*; FP: *P. australis* in abandoned farmland; PP: *P. australis* in drainage area of paddy field). Different letters after the values indicate statistically significant differences between five habitats in the same row. LSD: use ”least significant difference” as a method when test the differences between variables.

Photosynthetic Physiological Characteristics	HP	XP	SP	FP	PP
Im	1046.9 ± 12.3a	1118.7 ± 5.7a	1261.3 ± 11.2b	2165.3 ± 21.7c	2278.1 ± 20.3d
AQE	0.029 ± 0.004a	0.021 ± 0.003a	0.039 ± 0.002b	0.047 ± 0.004c	0.031 ± 0.003a
Rd	−0.65 ± 0.02a	−0.45 ± 0.01b	−0.55 ± 0.03c	−0.45 ± 0.07b	−0.29 ± 0.02d
Pmax	11.70 ± 0.13a	11.30 ± 0.25b	13.00 ± 0.24c	9.30 ± 0.11d	19.60 ± 0.17e
Bm	137.0 ± 2.7a	133.3 ± 3.5b	144.5 ± 5.4c	75.2 ± 6.8d	218.3 ± 5.9e

Note: Means (*n* = 10) followed by different letters are significantly different by LSD (*p* < 0.05).

**Table 2:** Photosynthetic physiological characteristics of *P. australis* in the breeding season (Im: saturated light intensity; AQE: apparent quantum efficiency; Rd: rate of dark respiration; Pmax: maximum net photosynthetic rate; Bm: biomass; HP: hygrophilous type of *P. australis*; XP: xerophytic type of *P. australis*; SP: psammophytic type of *P. australis*; FP: *P. australis* in abandoned farmland; PP: *P. australis* in drainage area of paddy field). Different letters after the values indicate statistically significant differences between five habitats in the same row. LSD: use ”least significant difference” as a method when test the differences between variables.

Photosynthetic Physiological Characteristics	HP	XP	SP	FP	PP
Im	2086.7 ± 11.4a	1810.2 ± 4.9b	1838.3 ± 11.9c	2186.3 ± 12.5d	924.1 ± 9.2e
AQE	0.011 ± 0.003a	0.017 ± 0.004b	0.033 ± 0.003c	0.007 ± 0.002d	0.025 ± 0.005e
Rd	−0.15 ± 0.06a	−0.14 ± 0.03a	−0.05 ± 0.02b	−0.35 ± 0.01c	−0.20 ± 0.04d
Pmax	11.50 ± 0.26a	14.30 ± 0.19b	19.50 ± 0.27c	9.00 ± 0.14d	9.50 ± 0.24d
Bm	448.3 ± 3.1a	250.0 ± 2.3b	166.7 ± 7.4c	365.0 ± 8.6d	665.3 ± 7.5e

Note: Means (*n* = 10) followed by different letters are significantly different by LSD (*p* < 0.05).

**Table 3:** Characteristics of CO_2_ response curve of *P. australis* in the growing season (Cm: CO_2_ saturation point; Cc: CO_2_ compensation point; φCO_2_: the highest quantum efficiency of CO_2_; Rl: rate of respiration; Pa: photosynthetic capacity; HP: hygrophilous type of *P. australis*; XP: xerophytic type of *P. australis*; SP: psammophytic type of *P. australis*; FP: *P. australis* in abandoned farmland; PP: *P. australis* in drainage area of paddy field). Different letters after the values indicate statistically significant differences between five habitats in the same row. LSD: use ”least significant difference” as a method when test the differences between variables.

Photosynthetic Parameters	HP	XP	SP	FP	PP
Cm	2195.7 ± 11.9a	1708.2 ± 4.0b	1678.9 ± 11.2c	3782.7 ± 12.0d	1334.9 ± 6.2e
Cc	13.8 ± 2.1a	12.5 ± 2.2b	6.3 ± 0.4c	48.6 ± 3.6d	12.2 ± 0.5b
φCO_2_	0.037 ± 0.004a	0.045 ± 0.002b	0.067 ± 0.001c	0.025 ± 0.001d	0.097 ± 0.005e
Rl	−0.50 ± 0.02a	−0.05 ± 0.01b	−0.40 ± 0.03a	−1.20 ± 0.02c	−1.30 ± 0.04c
Pa	26.70 ± 0.23a	26.30 ± 0.32b	26.60 ± 0.17a	28.10 ± 0.34c	30.70 ± 0.37d

Note: Means (*n* = 10) followed by different letters are significantly different by LSD (*p* < 0.05).

**Table 4:** Characteristics of CO_2_ response curve of *P. australis* in the breeding season (Cm: CO_2_ saturation point; Cc: CO_2_ compensation point; φCO_2_: the highest quantum efficiency of CO_2_; Rl: rate of respiration; Pa: photosynthetic capacity; HP: hygrophilous type of *P. australis*; XP: xerophytic type of *P. australis*; SP: psammophytic type of *P. australis*; FP: *P. australis* in abandoned farmland; PP: *P. australis* in drainage area of paddy field). Different letters after the values indicate statistically significant differences between five habitats in the same row. LSD: use ”least significant difference” as a method when test the differences between variables.

Photosynthetic Parameters	HP	XP	SP	FP	PP
Cm	3417.9 ± 11.2a	2791.7 ± 4.6b	1486.4 ± 11.2c	5465.8 ± 17.5d	2363.1 ± 9.0e
Cc	12.9 ± 0.7a	15.2 ± 1.3b	5.9 ± 0.3c	59.7 ± 8.8d	35.4 ± 2.7e
φCO_2_	0.021 ± 0.003a	0.039 ± 0.002b	0.017 ± 0.003c	0.011 ± 0.002d	0.049 ± 0.003e
Rl	−0.05 ± 0.006a	−1.1 ± 0.05b	−0.85 ± 0.03c	−0.65 ± 0.03d	−1.75 ± 0.1e
Pa	25.10 ± 0.70a	28.60 ± 0.23b	28.70 ± 0.37b	26.20 ± 0.45c	29.40 ± 0.68d

Note: Means (*n* = 10) followed by different letters are significantly different by LSD (*p* < 0.05).

**Table 5:** Characteristics of photosynthetic dark reaction of *P. australis* in the growing season (Vcmax: maximum carboxylation rate; Jmax: maximum electron transfer rate; Ci_transition: intersection point of the Vc-limit curve (blue) and J-limit curve (red); HP: hygrophilous type of *P. australis*; XP: xerophytic type of *P. australis*; SP: psammophytic type of *P. australis*; FP: *P. australis* in abandoned farmland; PP: *P. australis* in drainage area of paddy field). Different letters after the values indicate statistically significant differences between five habitats in the same row. LSD: use ”least significant difference” as a method when test the differences between variables.

Photosynthetic Parameters	HP	XP	SP	FP	PP
Vcmax	94.53 ± 2.61a	41.47 ± 2.04b	53.76 ± 3.23c	70.59 ± 1.62d	138.99 ± 3.93e
Jmax	148.28 ± 2.51a	107.29 ± 1.32b	111.57 ± 3.91b	115.13 ± 2.10b	195.75 ± 2.85c
Ci_transition	530	787	519	828	308

Note: Means (*n* = 10) followed by different letters are significantly different by LSD (*p* < 0.05).

**Table 6:** Characteristics of photosynthetic dark reaction of *P. australis* in the breeding season (Vcmax: maximum carboxylation rate; Jmax: maximum electron transfer rate; Ci_transition: intersection point of the Vc-limit curve (blue) and J-limit curve (red); HP: hygrophilous type of *P. australis*; XP: xerophytic type of *P. australis*; SP: psammophytic type of *P. australis*; FP: *P. australis* in abandoned farmland; PP: *P. australis* in drainage area of paddy field). Different letters after the values indicate statistically significant differences between five habitats in the same row. LSD: use ”least significant difference” as a method when test the differences between variables.

Photosynthetic Parameters	HP	XP	SP	FP	PP
Vcmax	86.91 ± 6.54a	61.85 ± 2.26b	94.26 ± 3.19c	30.21 ± 0.89d	82.90 ± 1.69a
Jmax	120.02 ± 4.77a	116.62 ± 3.53a	154.89 ± 3.08b	59.58 ± 1.43c	146.62 ± 2.19b
Ci_transition	405	815	428	999	808

Note: Means (*n* = 10) followed by different letters are significantly different by LSD (*p* < 0.05).

**Table 7:** Characteristics of five *P. australis* habitats.

Habitats	Density of Reed	Water Level (cm)	Soil Moisture (%)	Area (km^2^)
HP	131	20–40	43.12	2.48
XP	179	0	36.25	6.35
SP	25	0	18.29	2.93
FP	54	0	29.33	3.23
PP	126	30–60	54.72	2.87
